# Supplementary material for: A somatic piRNA pathway in the Drosophila fat body ensures metabolic homeostasis and normal lifespan
Source: Nat Commun. 2016 Dec 21;7:13856. doi: 10.1038/ncomms13856 (PMC5187580; doi:10.1038/ncomms13856)
Supplement: Supplementary Information — Supplementary Figures and Supplementary Tables. [file ncomms13856-s1.pdf]

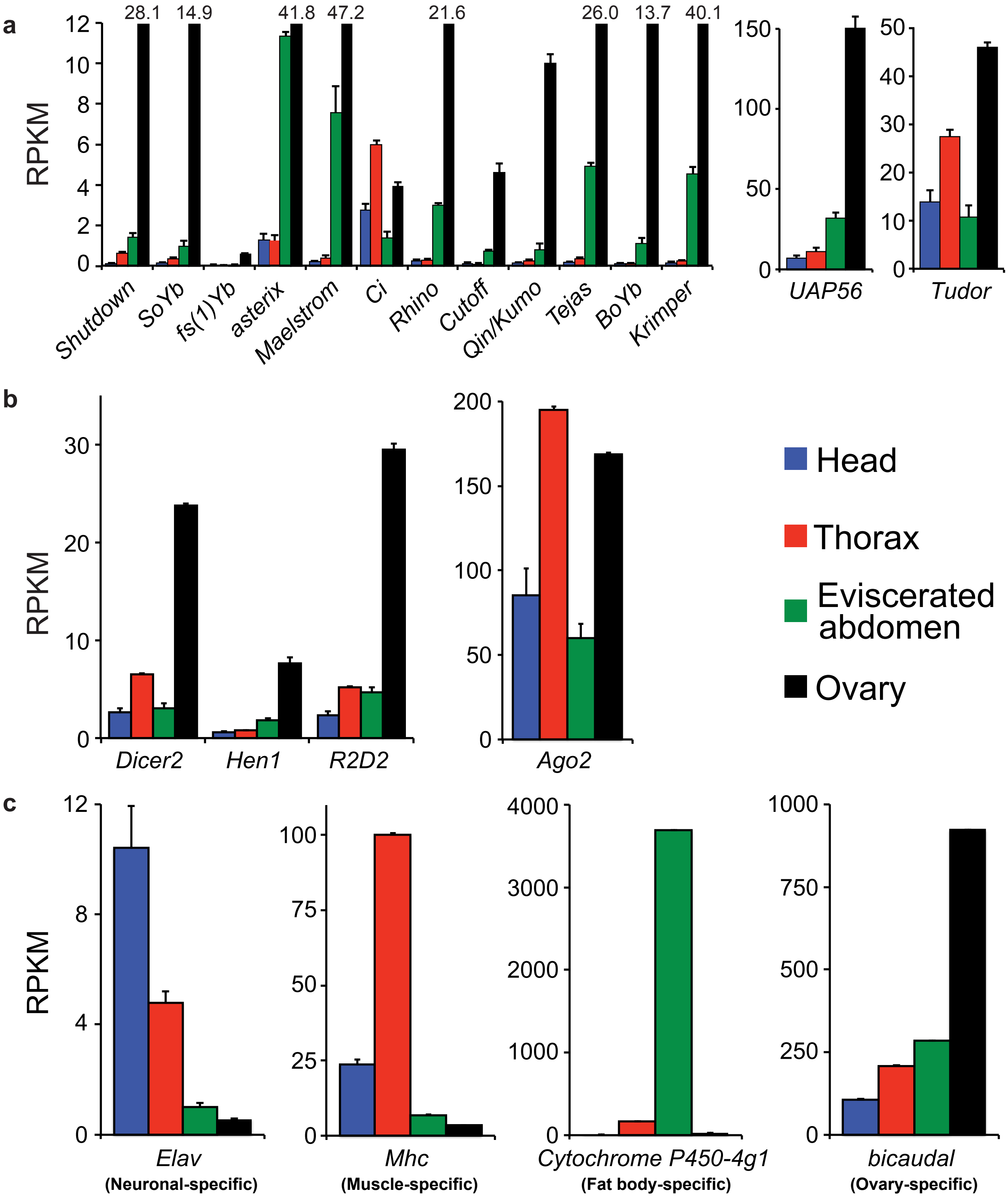


**Supplementary Figure 1: Gene expression profiles of somatic and reproductive tissues.**
(**a**) Expression of additional piRNA pathway genes generated from total RNA-seq libraries of head, thorax, eviscerated abdomen, and ovary. Data values for ovary libraries that exceed the range of the plot are shown above each relevant bar.
(**b**) Expression of siRNA pathway genes.
(**c**) Expression of tissue-specific control genes.
RPKM: Reads Per Kilobase per Million. Error bars represent S.E.M. $n$=3 replicate libraries. Of the 14 genes presented, 11 are statistically significantly increased in eviscerated abdomen compared to the head and thorax, one is significantly lower in eviscerated abdomen vs. thorax (*Ci*), and two are not significantly different (*fs(1)Yb* and *Tudor*); $P$<0.0001. See Supplementary Dataset 1 for relevant statistics.

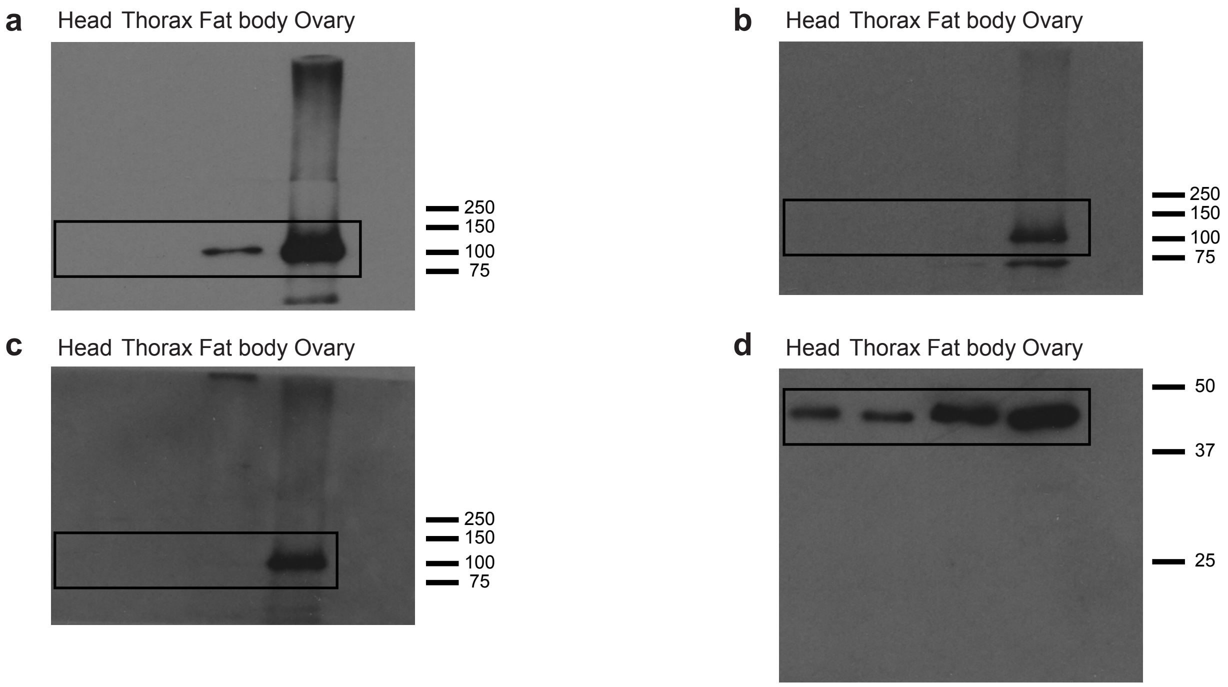


**Supplementary Figure 2: Original immunoblots used in Figure 1.**
Immunoblots staining for anti-Piwi (**a**), anti-AGO3 (**b**), anti-Aub (**c**), and anti-Actin (**d**). Black blocks indicate the bands selected for the main figure.

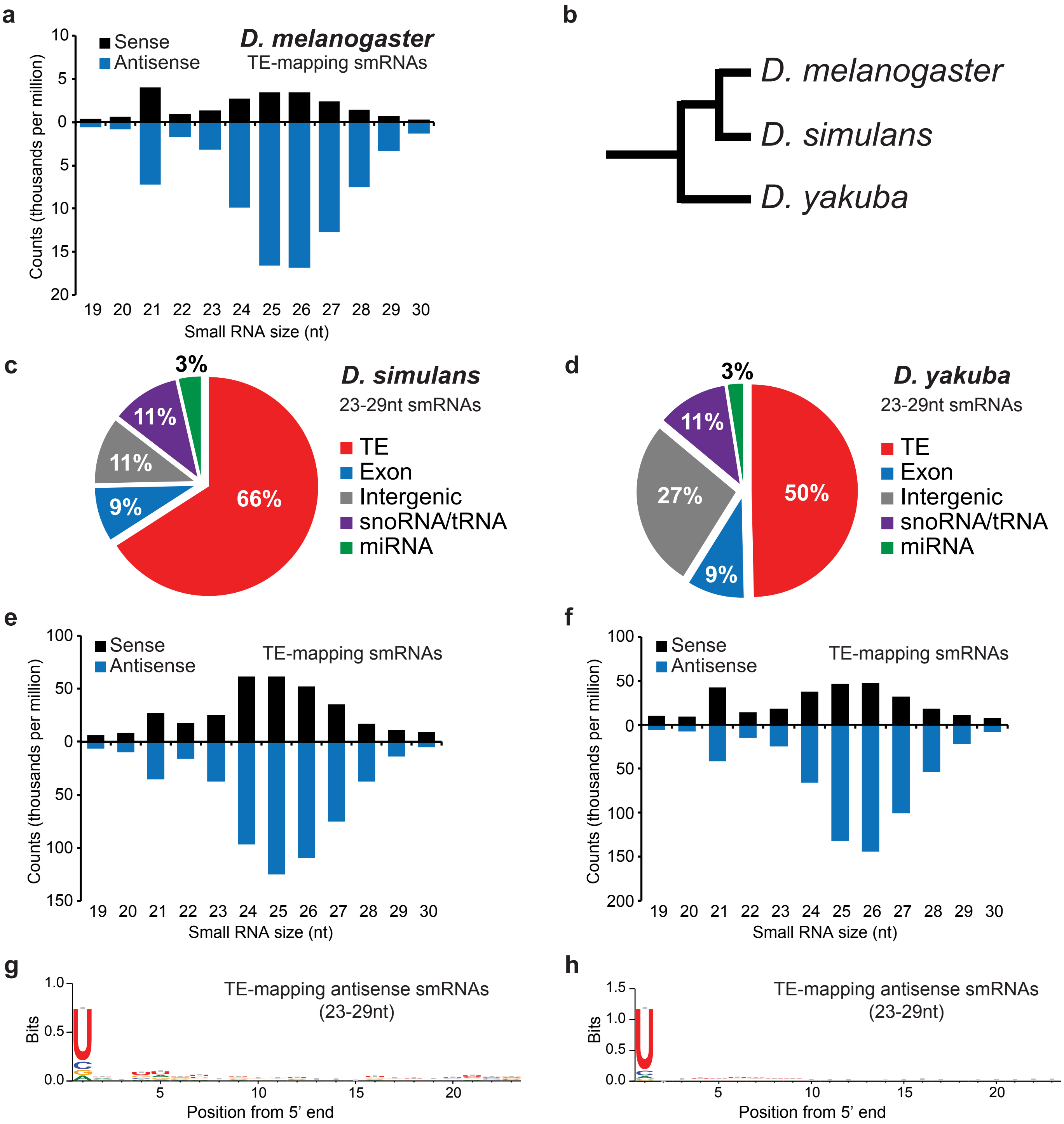


**Supplementary Figure 3: Fat body piRNA pathway signatures are conserved across drosophilid species.**

(**a**, **e**, **f**,) Fat body smRNA size profile of TE-mapping reads from oxidized smRNA-seq libraries (see **Fig. 1d**) of *D. melanogaster* (**a**), *D. simulans* (**e**), and *D. yakuba* (**f**). Reads are divided by mapping either sense (black) or antisense (blue) to TEs. Peak at 21nt likely represents siRNA population. Broader peak from 23-29nt represents putative fat body piRNAs.

(**b**) Schematic cladogram depicting the evolutionary relationships between three drosophilid species.

(**c**, **d**) Putative fat body piRNAs (23-29nt) aligned to the *D. simulans* (**c**) and *D. yakuba* (**d**) genomes map primarily to TEs.

(**g**, **h**) Sequence composition of TE-mapping putative fat body piRNAs (23-29nt) from *D. simulans* (**g**) and *D. yakuba* (**h**) displays a first position nucleotide bias for uracil.

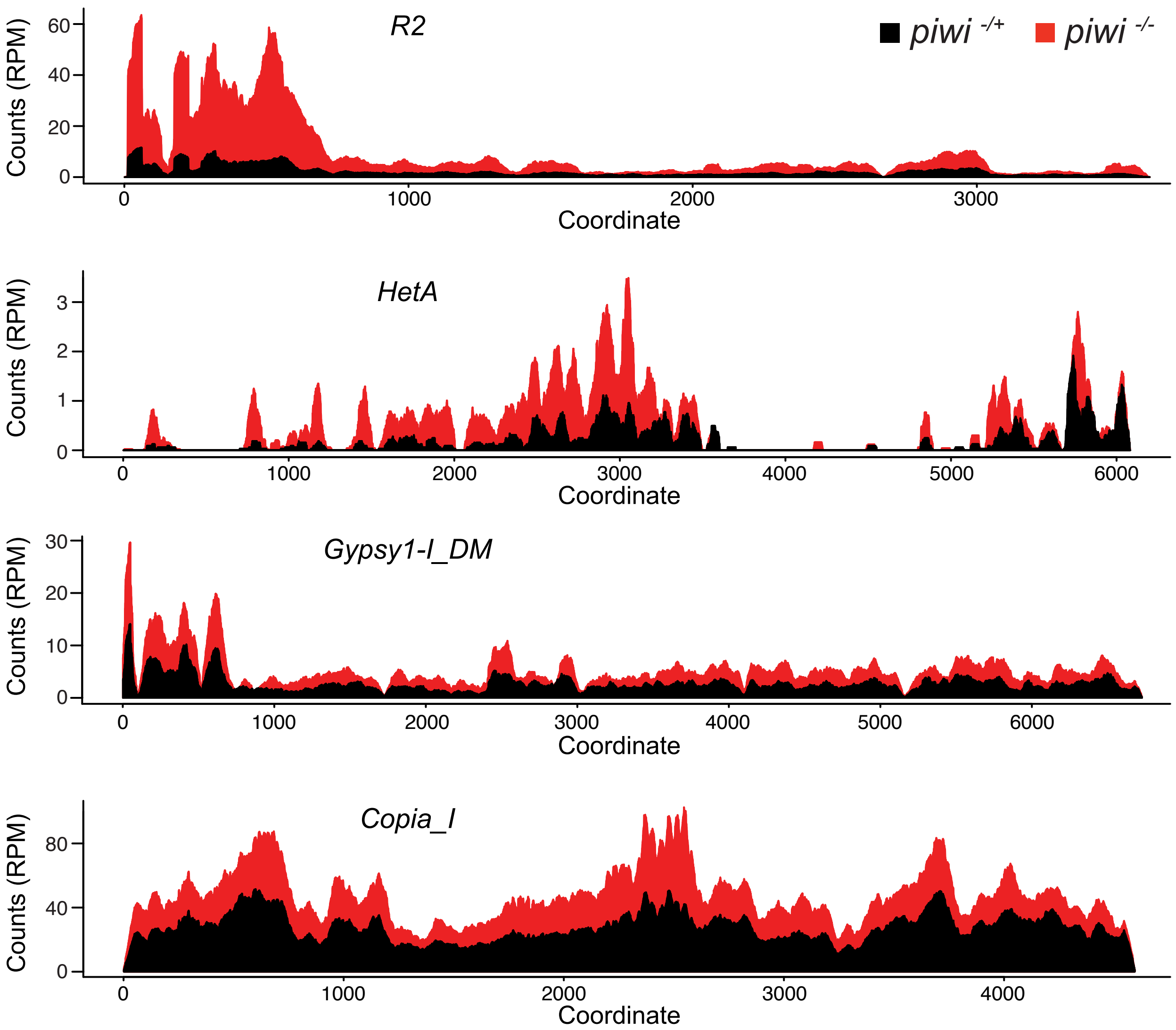


**Supplementary Figure 4: Expression profiles of representative upregulated TEs in *piwi* mutant and control fat bodies.**
*piwi* mutants show increased expression of TEs relative to heterozygous controls. Reads from *piwi* mutants (red) and controls (black) are shown spanning the consensus sequences of four representative TEs from **Fig. 2a**. RPM: Reads Per Million. $n$=3 replicate libraries.

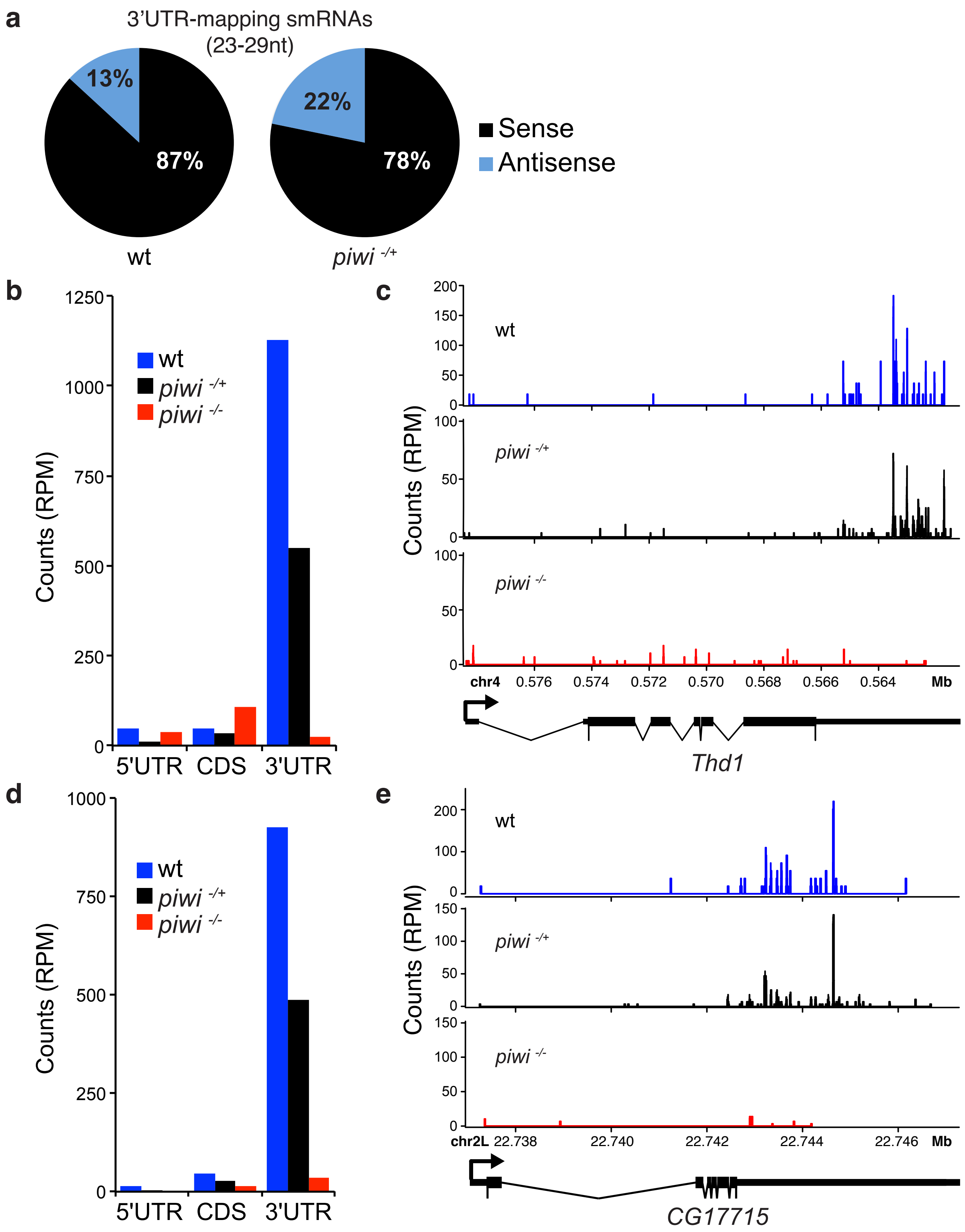


**Supplementary Figure 5: Characteristics of 3'UTR-mapping fat body piRNAs.**

(**a**) 3'UTR-mapping piRNAs (23-29nt) in wt and *piwi* $^{-/+}$ fat bodies are predominantly sense to coding genes.

(**b-e**) Representative genes featuring abundant sense 3'UTR-mapping piRNAs. Unique piRNA reads (23-29nt) map to the *Thd1* (**b**, **c**) and *CG17715* (**d**, **e**) gene bodies in wt and *piwi* heterozygotes and are lost in *piwi* mutants. Thick lines in gene model represent coding sequence; thin lines represent 5' and 3'UTRs. RPM: Reads Per Million. See Supplementary Dataset 2 for additional fat body genic piRNAs.

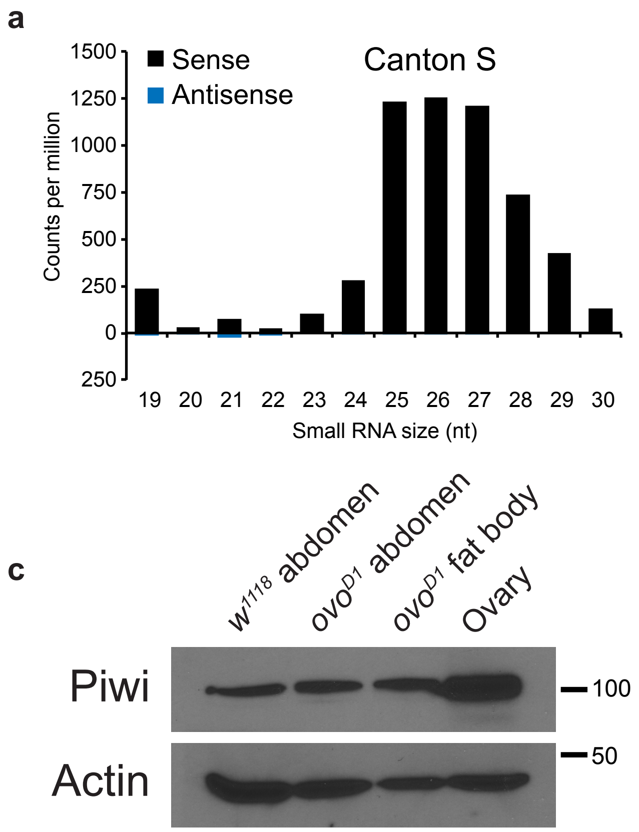


**Supplementary Figure 6: Flies lacking ovaries maintain fat body piRNA signatures.** (**a**, **b**) Size profile of small RNA-seq reads from purified fat body mapping uniquely to *flamenco* locus in wild-type Canton S flies (**a**) and $ovo^{D1}$ mutant flies lacking ovaries (**b**). Reads were normalized to total uniquely aligning reads in each library.

(**c**) Piwi protein is present in the eviscerated abdomens and pure fat bodies of $ovo^{D1}$ mutant flies lacking ovaries. Wild-type $w^{1118}$ eviscerated abdomens and ovaries serve as positive controls. Actin serves as a loading control.

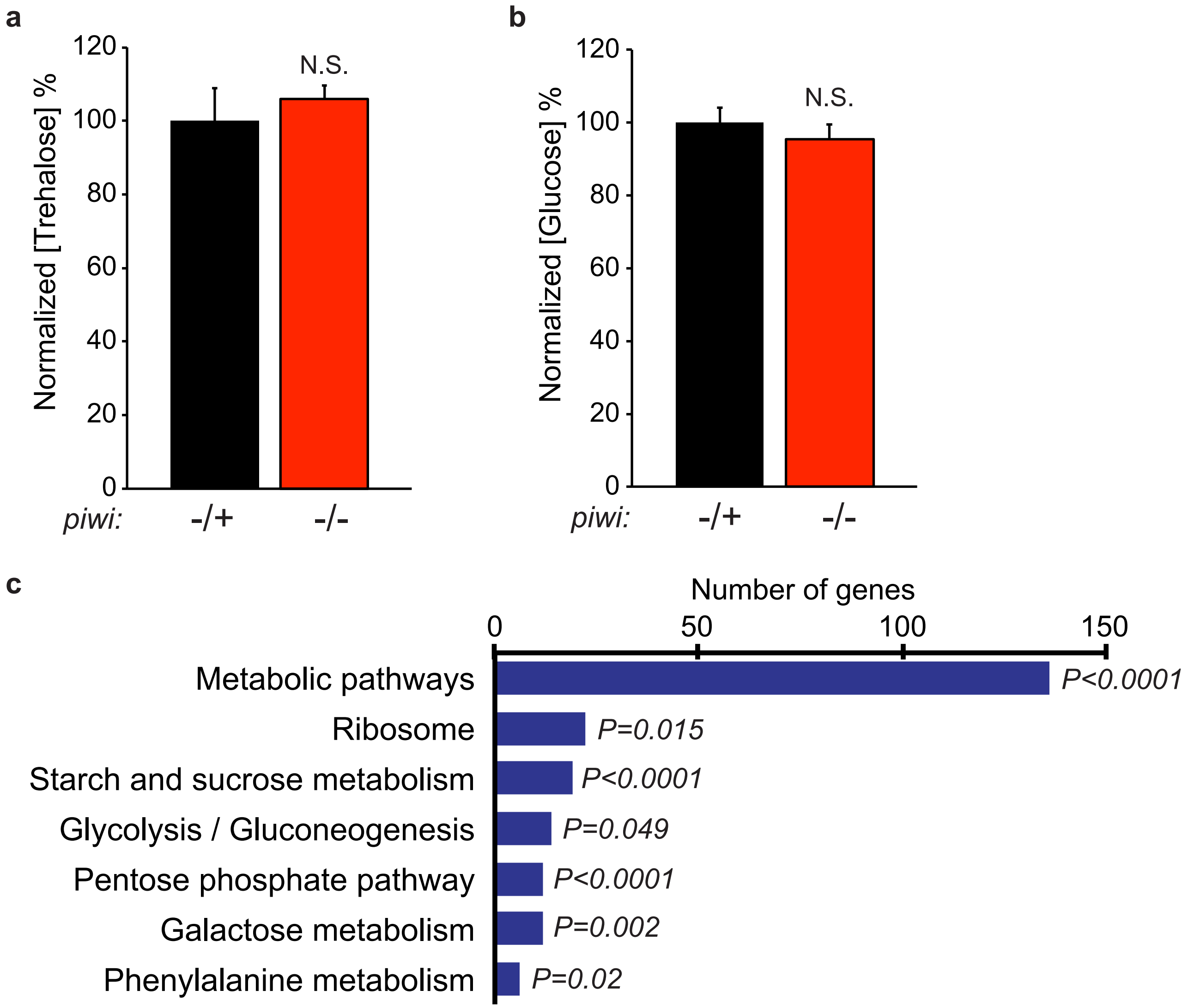


**Supplementary Figure 7: Metabolic phenotypes of *piwi* mutants.**
(**a**, **b**) Measurements of whole-body adult fly trehalose (**a**), and glucose (**b**) of *piwi* mutants compared to heterozygous controls. Data were normalized to total protein concentration of each sample and represented as a percent of the heterozygous control. Error bars are S.E.M. For each assay, *n*=5 biological replicates per genotype.
(**c**) KEGG pathway analysis of differentially expressed coding genes in fat body of *piwi* mutants compared to heterozygous controls. *n*=3 replicate libraries.

| *piwi* Starvation - 092414 | | Hours at % Mortality | | vs. (-/+) Control | |
|---|---|---|---|---|---|
| Genotype | *n*=X | 50% | 90% | $X^2$ | *P*-value |
| *piwi* $^{-/+}$ | 48 | 114 | 132 | — | — |
| *piwi* $^{-/-}$ | 48 | 48 | 66 | 77.81 | *P*<0.0005 |

| *piwi* Starvation - 100614 | | Hours at % Mortality | | vs. (-/+) Control | |
|---|---|---|---|---|---|
| Genotype | *n*=X | 50% | 90% | $X^2$ | *P*-value |
| *piwi* $^{-/+}$ | 100 | 114 | 138 | — | — |
| *piwi* $^{-/-}$ | 97 | 66 | 84 | 131.39 | *P*<0.0005 |

| *piwi* Immune Challenge - 070215 | | Days at % Mortality | | vs. Respective (-/+) Control | | vs. Respective (-) Control | |
|---|---|---|---|---|---|---|---|
| Genotype/Condition | *n*=X | 50% | 90% | $X^2$ | *P*-value | $X^2$ | *P*-value |
| *piwi* $^{-/+}$ (-) | 50 | N/A | N/A | — | — | — | — |
| *piwi* $^{-/-}$ (-) | 47 | N/A | N/A | 1.35 | N.S. | — | — |
| *piwi* $^{-/+}$ (+) | 50 | N/A | N/A | — | — | 0.21 | N.S. |
| *piwi* $^{-/-}$ (+) | 53 | 9 | N/A | 31.08 | *P*<0.0005 | 18.07 | *P*<0.0005 |

| *piwi* Immune Challenge - 073115 | | Days at % Mortality | | vs. Respective (-/+) Control | | vs. Respective (-) Control | |
|---|---|---|---|---|---|---|---|
| Genotype/Condition | *n*=X | 50% | 90% | $X^2$ | *P*-value | $X^2$ | *P*-value |
| *piwi* $^{-/+}$ (-) | 50 | N/A | N/A | — | — | — | — |
| *piwi* $^{-/-}$ (-) | 50 | N/A | N/A | 17.43 | *P*<0.0005 | — | — |
| *piwi* $^{-/+}$ (+) | 50 | N/A | N/A | — | — | 6.32 | *P*=0.0119 |
| *piwi* $^{-/-}$ (+) | 50 | 10 | N/A | 23.56 | *P*<0.0005 | 14.74 | *P*<0.0005 |

| *piwi* Lifespan - 080914 | | Days at % Mortality | | vs. (-/+) Control | |
|---|---|---|---|---|---|
| Genotype | *n*=X | 50% | 90% | $X^2$ | *P*-value |
| *piwi* $^{-/+}$ | 254 | 67 | 75 | — | — |
| *piwi* $^{-/-}$ | 245 | 29 | 51 | 389.06 | *P*<0.0005 |

| *piwi* Lifespan - 072414 | | Days at % Mortality | | vs. (-/+) Control | |
|---|---|---|---|---|---|
| Genotype | *n*=X | 50% | 90% | $X^2$ | *P*-value |
| *piwi* $^{-/+}$ | 238 | 66 | 72 | — | — |
| *piwi* $^{-/-}$ | 225 | 30 | 56 | 314.39 | *P*<0.0005 |

| *flam* Lifespan - 040815 | | Days at % Mortality | | vs. (-/+) Control | |
|---|---|---|---|---|---|
| Genotype | *n*=X | 50% | 90% | $X^2$ | *P*-value |
| *flam* $^{-/+}$ | 250 | 58 | 68 | — | — |
| *flam* $^{-/-}$ | 245 | 44 | 56 | 226.93 | *P*<0.0005 |

| *flam* Lifespan - 051615 | | Days at % Mortality | | vs. (-/+) Control | |
|---|---|---|---|---|---|
| Genotype | *n*=X | 50% | 90% | $X^2$ | *P*-value |
| *flam* $^{-/+}$ | 263 | 58 | 68 | — | — |
| *flam* $^{-/-}$ | 258 | 42 | 58 | 221.99 | *P*<0.0005 |

| *flam* $^{-/-}$ 3TC Lifespan - 012816 | | Days at % Mortality | | vs. EtOH Control | |
|---|---|---|---|---|---|
| Genotype/Condition | *n*=X | 50% | 90% | $X^2$ | *P*-value |
| *flam* $^{-/-}$ (Control) | 240 | 47 | 61 | — | — |
| *flam* $^{-/-}$ (3TC) | 251 | 55 | 67 | 22.48 | *P*<0.0005 |

| *flam* $^{-/-}$ 3TC Lifespan - 051916 | | Days at % Mortality | | vs. EtOH Control | |
|---|---|---|---|---|---|
| Genotype/Condition | *n*=X | 50% | 90% | $X^2$ | *P*-value |
| *flam* $^{-/-}$ (Control) | 246 | 47 | 71 | — | — |
| *flam* $^{-/-}$ (3TC) | 254 | 57 | 67 | 16.97 | *P*<0.0005 |

**Supplementary Table 1: Parameters and statistical analysis of starvation, immune challenge, and lifespan assays.**
